# Damaged Proseal™ LMA inflation line can be repaired

**DOI:** 10.4103/0019-5049.71029

**Published:** 2010

**Authors:** Pankaj Kundra, Bhaskar Nisha

**Affiliations:** Department of Anaesthesiology and Critical Care, Jawaharlal Institute of Postgraduate Medical Education and Research, Pondicherry - 605 006, India

Sir,

Laryngeal mask airway (LMA) Proseal™ is available as a reusable device made up of medical grade silicone that can be autoclaved 40 times.[1,2] Its life span depends on the number, temperature and duration of the autoclave cycles. However, these devices can also be damaged during clinical use from biting,[3] surgical instruments, accidental introduction of fluid into the cuff[4] and during cleaning and disinfection.[2]

**Figure 1:**
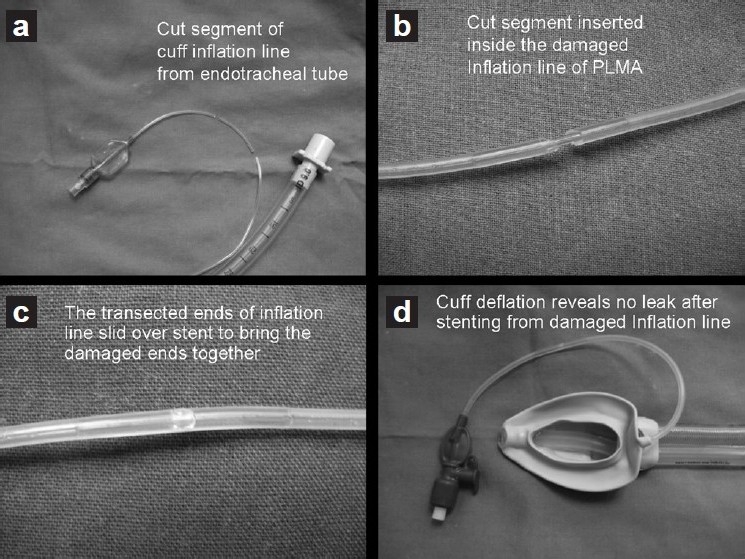
Steps of repair of the inflation line

A size 3 LMA Proseal™ was discovered with a damaged inflation line during routine morning inspection before use. The damage was incurred as the inflation line was caught between the lid and the metal box during storage. The cuff and shaft of the LMA were intact, as confirmed by close visual inspection and by placing it under water after full inflation. Because it was an isolated damage to the inflation line, the possibilities of repairing the inflation line were explored to put it back to use without jeopardizing patient safety. A 4-cm cut-segment of the inflation line of a 9-mm (internal diameter) polyvinyl chloride (PVC), disposable, cuffed endotracheal tube was used as an internal stent to oppose the transected ends of the inflation line of the Proseal™ LMA. The internal diameter of the Proseal™ LMA inflation line (1.5 mm) provided a snug fit on the cut segment of the endotracheal tube pilot balloon inflation line with an external diameter of 1.7 mm without the use of an adhesive [Figure 1]. The Proseal™ LMA was autoclaved and its integrity was confirmed to ensure there was no leak before putting it to clinical use. Since repair, the Proseal™ LMA has already been put to use 12 times, and it has withstood autoclaving satisfactorily.

Given the expensive capital cost of reusable Proseal™ LMA (Rs. 12,000 per piece approximately), a repair can be attempted if an isolated damage to the inflation line is discovered. However, care must be taken to ascertain that other components are intact and a preuse safety test should be performed before using them on patients.
